# Influence of Various Technologies on the Quality of Ultra-Wideband Antenna on a Polymeric Substrate

**DOI:** 10.3390/polym14030507

**Published:** 2022-01-27

**Authors:** Peter Lukacs, Alena Pietrikova, Igor Vehec, Peter Provazek

**Affiliations:** Department of Technologies in Electronics, Faculty of Electrical Engineering and Informatics, Technical University of Kosice, Park Komenskeho 2, 040 01 Kosice, Slovakia; alena.pietrikova@tuke.sk (A.P.); igor.vehec.2@tuke.sk (I.V.); peter.provazek@tuke.sk (P.P.)

**Keywords:** polyimide film, polymer thick-film technology, screen-printing, inkjet-printing, UWB antenna

## Abstract

The design, simulation, realization, and measurement of an ultra-wideband (UWB) antenna on a polymeric substrate have been realized. The UWB antenna was prepared using conventional technology, such as copper etching; inkjet printing, which is regarded as a modern and progressive nano-technology; and polymer thick-film technology in the context of screen-printing technology. The thick-film technology-based UWB antenna has a bandwidth of 3.8 GHz, with a central frequency of 9 GHz, and a frequency range of 6.6 to 10.4 GHz. In addition to a comparison of the technologies described, the results show that the mesh of the screens has a significant impact on the quality of the UWB antenna when utilizing polymeric screen-printing pastes. Last but not least, the eco-friendly combination of polyimide substrate and graphene-based screen-printing paste is thoroughly detailed. From 5 to 9.42 GHz, the graphene-based UWB antenna achieved a bandwidth of 4.42 GHz. The designed and realized UWB antenna well exceeds the Federal Communications Commission’s (FCC) standards for UWB antenna definition. The modification of the energy surface of the polyimide substrate by plasma treatment is also explained in this paper, in addition to the many types of screen-printing pastes and technologies. According to the findings, plasma treatment improved the bandwidth of UWB antennas to 5.45 GHz, and the combination of plasma treatment with graphene provides a suitable replacement for traditional etching technologies. The characteristics of graphene-based pastes can also be altered by plasma treatment in terms of their usability on flexible substrates.

## 1. Introduction

Ultra-wideband is a comprehensive term that refers to short-range wireless technology that consumes low power and transmits data over a wide bandwidth (more than 500 MHz or with a relative bandwidth greater than 20%) in the frequency range of 3.1 to 10.6 GHz [1]. A high transmission rate can be achieved by combining a large bandwidth covering the majority of the radio spectrum with an appropriate modulation (such as Pulse Position Modulation (PPM), Pulse Amplitude Modulation (PAM), Bi-Phase Modulation (BPSK), Code Division Multiple Access (CDMA), or Orthogonal Frequency Division Multiplexing (OFDM)) [2]. UWB technology has a wide range of applications, including data transmission and sensor data collecting in modern networks, such as WPAN (Wireless Personal Area Networks) for interconnecting electronic devices [2] and WBAN (Wireless Body Area Networks) for continuous health monitoring [3]. Additionally, it can be utilized to locate or detect moving objects, such as in collision avoidance systems or human sensing.

Patch antennas are a common type of UWB antenna. Circular, elliptical, triangular, rectangular, or polygonal are the most basic shapes of patch antennas [4]. However, the variety of patch antenna topologies is enormous at the moment and far from being limited to these fundamental designs. Patch antennas are frequently taking on more complicated shapes and even fractal patterns in order to increase their parameters and efficiency [5,6]. Various structures, such as the Defected Ground Structure (DGS), can also be used to improve the parameters of antennas [7]. The antenna’s impedance matching with the feed line, which is frequently a microstrip, is a significant parameter. Advanced methods such as the Genetic Algorithm (GA), Particle Swarm Optimization (PSO), and Differential Evolution (DE) can also be utilized to optimize the topology of patch antennas and antenna arrays [8,9]. As a result, contemporary design processes are intimately connected to Computer-Aided Design (CAD), simulation, and computational tools. However, even with the best design, simulation, and optimization techniques, if the limitations of the manufacturing technology are not considered, the output is frequently worthless.

Subtractive and additive processes can be used to fabricate UWB antennas on rigid or flexible substrates. Subtractive methods are frequently used to manufacture UWB antennas on rigid substrates. Typically, they have printed circuit boards (PCBs) with a variety of insulating materials (based on typical glass-reinforced epoxy laminates, hydrocarbon ceramic laminates, polytetrafluoroethylene, etc.) and copper foil cladding. They cover a diverse range of antennas intended for a variety of purposes. The fundamental additive techniques utilized in patch antenna fabrication include screen printing, gravure printing, inkjet printing, and micro-dispensing [10]. Screen printing is a well-established and widely used technique for thick-film deposition. Using polymeric thick-film (PTF) technology enables the use of both rigid and flexible organic substrates due to their low curing temperatures. When it comes to the deposition of conductive layers, they are typically inks or pastes based on silver, carbon, or graphene materials. While the inkjet printing method also benefits from low sintering temperatures, there is a substantial difference in the much thinner thickness of the deposited layer (less than 1 µm) in comparison to PTF (usually a few µm to 20 µm). This also involves stricter requirements for the flatness, roughness, and surface quality of the substrate. Patch antennas have been manufactured using silver- or graphene-based materials via screen printing [11,12] or inkjet printing on a variety of substrates, including paper [13], polyimide [14,15], or glass [16]. Screen printing [17,18,19], inkjet printing [20], or a combination of these printing methods [21] can also be utilized to create wearable antennas in the current era. Due to the strict limitations and needs for green electronics, biodegradable and environmentally friendly materials are increasingly being used in the field of antennas [22,23]. There are substrate materials such as cellulose-acetate (CA) [24], glucopyranoside (GPTE), and diethylene-toluene-tetramine (DETDA) [25] that can be used in place of conventional polymeric substrates. The surface quality of polymeric substrates is a critical technological factor in the HF area [26].

Certain applications, such as WBAN, depend on wearable UWB antennas to function [27]. There are numerous technologies available for producing UWB antennas in terms of wearable electronics. For example, a non-conductive fabric (jeans, fleece, felt, plain-woven polyester fabric, etc.) can be utilized to fabricate a wearable antenna with a radiating component made of conductive materials, such as copper foil [28,29] or another conductive fiber [30,31]. Even wearable antennas made from various conductive threads have been investigated, whether they are made of electroconductive polyester yarn covered in Cu–Ni alloy [32] or a silver-plated nylon thread [33]. For antennas on flexible substrates, and particularly for wearable antennas, it is necessary to take into account the effect of bending or crumpling the substrate with the patch antenna on the resulting parameters, such as the antenna resonant frequency, scattered parameters, radiation pattern, and S_11_ parameter [34,35,36], as well as the mechanical properties of the substrate, especially for wearable antennas [37].

Numerous high-frequency characteristics are influenced by the electrical characteristics of the substrate dielectric material (such as dielectric constant, dielectric loss factor, or roughness). Increased loss factor (tanδ) and dielectric constant (ε_r_) of the substrate have a negative impact on signal transmission loss. Take note that frequency has an effect on signal transition loss as well. Increased dielectric constant values also slow down signal transmission and limit the antenna’s bandwidth. Both the dielectric loss factor and the dielectric constant are dependent on the polarization process and structure of the substrate material [38,39]. Surface characteristics, such as roughness, might also be important, particularly in a high-frequency application. The root-mean-square (RMS) roughness level of the substrate surface has an effect on impedance and scattering losses, which can result in additional attenuation on microstrip lines [40,41,42]. In antenna applications, surface roughness has a negative impact on antenna gain [43]. Additionally, metamaterials based on split-ring resonators (SRRs), complementary split-ring resonators (CSRRs), capacitance-loaded strips (CLSs), or similar structures can be used to improve high-frequency characteristics [44].

The roughness of the surface is also important technologically, as it affects the adhesion and wettability of deposited layers. While increased surface roughness improves the adhesion, it might cause issues when depositing thin films (such as in inkjet technology, where the thickness of a deposited layer is in the hundreds of nm range) [26].

The typical dielectric materials (Table 1), such as polyimide (PI), polytetrafluoroethylene (PTFE), polyethylene terephthalate (PET), polydimethylsiloxane (PDMS), or liquid crystal polymers (LCPs), and polypropylene (PP), have suitable characteristics for high-frequency applications.

Additionally, various additives, such as glass or ceramics, might improve dielectric characteristics [49,50]. The low glass transition temperature of polymer substrates can be a limiting factor in printed electronics production technologies that require the heat processing (such as curing or sintering) of conductive layers [51].

Polymer substrates currently dominate the field of flexible electronics [45] and make transparent structures possible [52]. Today’s trends in high-frequency applications, particularly in the GHz and THz bands, require the use of low-loss substrate materials that provide improved bandwidth performance and higher radiation efficiencies [40]. In general, a wide variety of polymeric substrate materials can meet all demanding requirements, making them an essential part of modern electronic technologies and occupying a significant role in them.

From the ecological point of view, the conventional method, which is based on subtractive etching on a metal-plated substrate (FR4, Pyralux), generates considerable amounts of metal, salt, and chemical waste products that are harmful to the environment [53]. These wastes must be disposed of according to legally required processes, significantly increasing the cost of production despite the process’s simplicity. This is one of the reasons why electronics trends are turning toward the use of eco-friendly and green manufacturing processes, and the use of polymeric and organic materials. As a result, this article compares conventional and modern polymer thick-film technologies by using silver and graphene-based materials in the context of UWB antenna realization. The novelty of this article is that it contains an investigation of the impact of a wide variety of technological factors, different kinds of technologies, and materials on the designed and realized UWB antenna. The paper discusses the effect of previously unstudied technological factors on the scattering parameter of an UWB antenna, such as the effect of the screen’s mesh on the reflection coefficient of an antenna, or the combination of conventional, thick- and thin-film technologies on a polymeric substrate. The results show that the combination of graphene-based screen-printing paste and a polyimide substrate is a potential alternative to conventional PCB technology for realizing UWB antennas.

## 2. Materials and Methods

The experiments can be divided into two steps. The first step is antenna design, which includes determining the antenna’s shape, calculating the radiation, feeding element dimensions, and simulating the proposed antenna. This stage is equally as critical as the second, which is focused on the antenna realization. To execute this, the designed antenna was realized on the polymeric polyimide substrate Kapton using three different technologies and three different conductive materials, as described in the following sections of this paper.

### 2.1. Antenna Design

**Figure 1:**
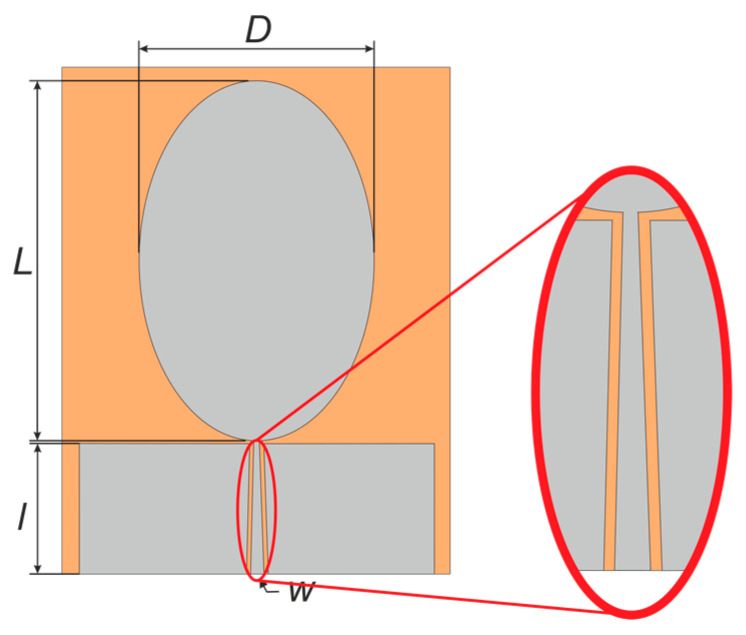
The proposed antenna with the critical point highlighted from the technological point of view.

The mathematical calculation of the important parts of the antenna, such as the diameter (D) and length (L) of the radiation element, as well as their ratio (D:L), is part of the UWB antenna design. In addition, the length (l) and width (w) of the feeding element are calculated in the design as well. The software Ansoft HFSS^TM^ was utilized for design and simulation purposes. Figure 1 shows the planned antenna with an elliptical coplanar waveguide shape.

The following equation can be used to compute the patch width W [14,54]:

(1) $$W=\frac{c}{2f_{l}\sqrt{\frac{\left(\varepsilon_{r}+1\right)}{2}}},$$

where ε_r_ presents the substrates’ dielectric constant, c indicates the light’s velocity in a free space.

The patch’s fringing fields can operate like a radiating slot. The extended length of patch ΔL is empirically determined like [54,55]:

(2) $$\Delta L=0.412h\frac{\left[\left(\varepsilon_{\mathrm{eff}}+0.3\right)\left(\left(\frac{W}{h}\right)+0.264\right)\right]}{\left[\left(\varepsilon_{\mathrm{eff}}-0.258\right)\left(\left(\frac{W}{h}\right)+0.8\right)\right]},$$

where h is the dielectric substrate’s height, W is the patch’s width, ε_eff_ is the effective dielectric constant and can be calculated by [54,56]:

(3) $$\varepsilon_{\mathrm{eff}}=\frac{\varepsilon_{r}+1}{2}+\frac{\varepsilon_{r}-1}{2}\left(\frac{1}{\sqrt{1+12\left(\frac{h}{W}\right)}}\right),$$

Thus, the length L of the parch can be calculated by [55]:

(4) $$L=L_{\mathrm{eff}}-2\Delta L,$$

where L_eff_ presents the effective length of the patch, and is given as [56]:

(5) $$L_{\mathrm{eff}}=\frac{c}{2f_{l}\sqrt{\varepsilon_{\mathrm{eff}}}},$$

The length L of the patch is given also as [56]:

(6) L=cfl2−2ΔL,

If the patch is elliptical in shape, the diameter D of an elliptical patch can be determined to get a more precise estimate of patch sizes as [54]:

(7) $$D=\frac{c}{f_{l}\sqrt{\varepsilon_{r}}},$$

The ratio R can be calculated as [54]:

(8) $$R=\frac{L}{D},$$

The impedance matching to 50 Ω was achieved by adjusting the length of the feeding element by [57]:

(9) $$l=\frac{\lambda }{4\sqrt{\varepsilon_{\mathrm{eff}}}},$$

The width of the feeding element can be estimated using the formula [57]:

(10) $$w=\frac{2h}{\pi }\left\{B-1-\ln\left(2B-1\right)+\frac{\varepsilon_{r}-1}{2\varepsilon_{r}}\left[\ln\left(B-1\right)+0.39-\frac{0.61}{\varepsilon_{r}}\right]\right\},$$

while,

(11) $$B=\frac{60\varepsilon_{r}^{2}}{Z_{0}\sqrt{\varepsilon_{r}}}\;$$

### 2.2. Samples Preparation

The primary goal of this paper is to investigate the impact of several traditional and advanced technologies on the quality of a designed UWB antenna in context of the scattering parameter S_11_. The polymeric substrate material used in all of the technologies investigated is the same.

The polyimide-based substrate DuPont^TM^ Kapton^®^ HN 200 was chosen as the isolation material due to its high temperature resistance (from −269 °C to 400 °C), suitable dielectric constant ε_r_ = 3.4, and low loss tangent δ = 0.002 [14]. According to the mathematical calculations, the thickness of DuPont^TM^ Kapton^®^ HN 200 is 50.8 µm and the dimensions of the polyimide foil used as an antenna substrate are 36 × 47.5 mm. Including the antenna design, various technological and material characteristics had to be evaluated in order to ensure the antenna’s practical realization, which has a substantial effect on the final quality.

Three technologies make use of copper plate, silver, and graphene particles in the form of nano-inks, and polymeric screen-printing pastes were used for the antenna realization. As a conventional technology, the standard wet etching process by the use of hydrochloric acid on the copper was utilized. For this reason, the Pyralux material was selected by regarding the thickness of the polyimide 50.8 µm and the two thicknesses of the copper plate (17.5 and 35 µm). In addition to conventional technology, inkjet printing and screen printing was applied as well. The PixDro PL50 inkjet printer was used with a Spectra^®^ SE-128 AA printhead and a nozzle diameter of 35 µm. The substrate was heated to 60 °C during the printing process. Throughout the printing process, the printhead remained at ambient temperature. The distance between the substrate and the nozzle was 0.5 mm, which eliminated the drop deflection and excessive drop spreading, both of which can have a negative impact on the antenna’s radiation. The conductive structure was printed using inkjet printing technology with Amepox’s silver-based nano-ink, JP-6n. The thickness of the printed conductive silver layer was around 1.15 µm, which was insufficient to generate a homogeneous structure. To ensure the structure’s electrical and mechanical properties were uniform, three layers of conductive silver nano-ink were printed onto the substrate. The silver layer printed onto the polyimide substrate has a final thickness of approximately 3.45 µm. The printed structure has to be reduced by 5% before printing due to ink spreading on the substrate during the deposition process. The printed structure was dried for 30 minutes at 60 °C and then sintered for 60 minutes at 220 °C in a typical laboratory oven.

Except for the inkjet printing technology, screen printing with three different pastes was also evaluated. Two of these are polymeric pastes filled with silver particles, Britrade’s XZ250 and Electra DOR’s ED3000, while the third is a graphene-based screen-printing paste, Electra DOR’s ED5020. The conductive paste ED3000 was created for use on untreated polyester, polyimide, and polycarbonate films. The XZ250 paste is a highly conductive silver-based paste designed for the fabrication of flexible printed circuit boards. The ED5020 is a conductive carbon-filled polymer paste that may be printed on a screen. The paste ED3000 has a sheet resistance of 30 mΩ/sq, the screen-printing paste XZ250 has a sheet resistance of 40 mΩ/sq, and the paste ED5020 has a sheet resistance of 20 Ω/sq, as stated in the datasheets.

To investigate the impact of these technological factors on the antennas’ quality, samples were manufactured by five various screen mashes (200, 250, 300, 325, and 400) using the automation screen-printer Aurel 900PA LTCC. It is necessary to keep in mind that different mashes result in a difference in the smoothness of the printed structure’s edges. This is critical in the high-frequency area, especially for antennas operating at GHz frequency bandwidths. After 10 minutes of drying at room temperature, the screen-printed structures were sintered according to the datasheets of the applied pastes in a typical laboratory oven (Memmert UNE 200), for 30 minutes at 120 °C for the XZ250 and ED3000 pastes, and 30 minutes at 150 °C for the ED5020 paste. The surfaces and edges of the printed antennas were inspected using the ZEISS Axio Observer optical microscope, Z1m.

To investigate the effect of direct discharge plasma on the quality of UWB antennas, the substrates were immediately treated with Piezobrush^®^ PZ3 before the screen-printing process. The CNC machine was used to control the Piezobrush’s movement. The Piezobrush’s speed was set to 1 cm/s, and the distance between the plasma and the substrate was set to 1 mm. Each substrate has been plasma treated twice.

## 3. Results and Discussion

The reflection or scattering parameter S_11_ was evaluated as a quality factor for the designed, manufactured, and measured UWB antenna. The red line represents the simulated reflection coefficient of the designed UWB antenna and acts as a reference curve in all graphs in this section. According to the simulation results, the designed antenna has a BW of 3.2 GHz, ranging from 7.35 GHz to 10.55 GHz. The reflection coefficient was measured using the Agilent N5241A PNA network analyzer. SMA connectors were used to connect the measurement cable to the antenna, which was conductively bonded to the antenna using the epoxy-based conductive glue NCNR 8331.

**Figure 2:**
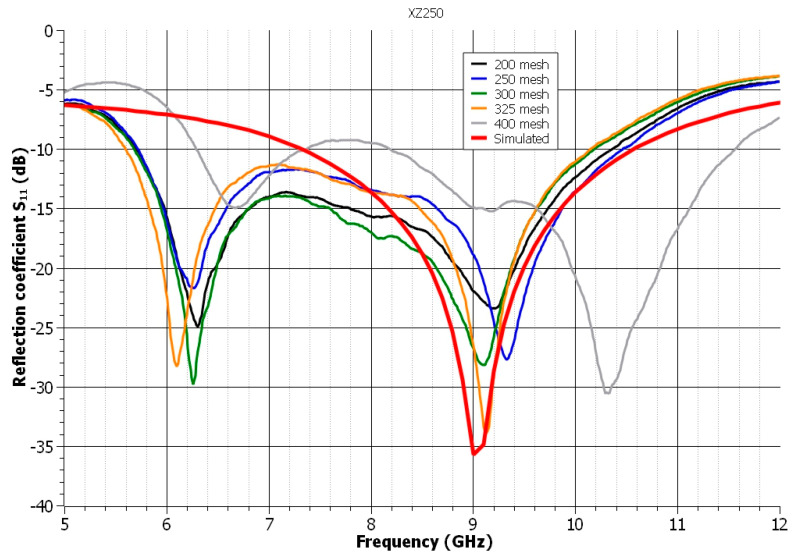
The influence of the mesh of the used screens on the reflection coefficient of the antenna—paste XZ250.

In Figure 2, the impact of the mesh of the used screens on the UWB antenna’s reflection coefficient is illustrated using the screen-printing paste XZ250. The worst results were obtained with 200 mesh due to the lowest attenuation. The best results in terms of resonance frequency and high attenuation were obtained when 325 mesh was used. The 400 mesh showed a significant shift in resonance frequency. Although the resonant frequency has been slightly moved to 9.15 GHz, the reflection coefficient is nearly identical to that of the simulated curve. The observed improvement in the measured S_11_ parameter was achieved when the mesh was increased from 200 to 325. According to the datasheet for the examined paste, the manufacturer recommends mesh sizes ranging from 124 to 228 (49–90 T/cm), which is much less than the mesh sizes used in the experiments. A smaller mesh size limits the creation of precise structures, but it is ideal for printing pastes with a higher viscosity and larger particles. Although a larger mesh is better for printing accurate structures, strict requirements for the rheological properties of the applied pastes exist. However, it has been demonstrated that this paste is suited for higher meshes, with results demonstrating that it is applicable up to 325 mesh and increases the quality of printed structures.

**Figure 3:**
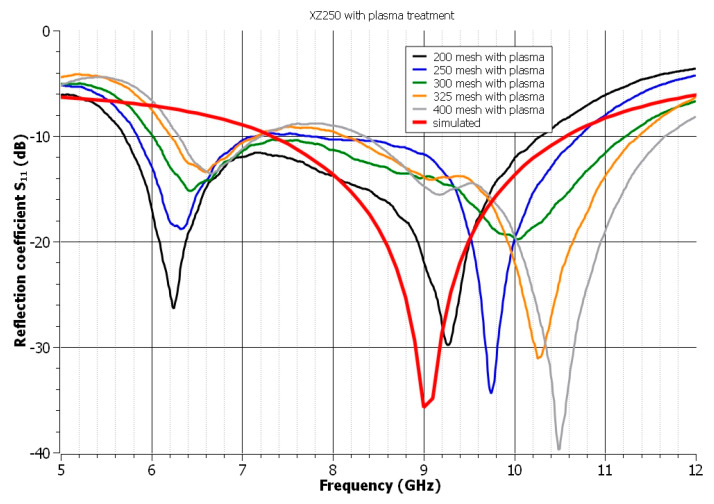
The influence of the mesh of the used screens on the reflection coefficient of the antenna after the plasma treatment—paste XZ250.

Plasma discharge treatment of the surface increases the surface free energy [58]. This leads to a change in the wettability of the polymeric substrates’ surfaces, which results in the spread of the screen-printing paste during the deposition process. As illustrated in Figure 3, the dimensions of the UWB antenna’s radiating and feeding element varied, resulting in an uncontrolled increase in the antenna’s resonant frequency. On the other hand, plasma treatment contributes to the smoothing of the printed structure’s edges.

**Figure 4:**
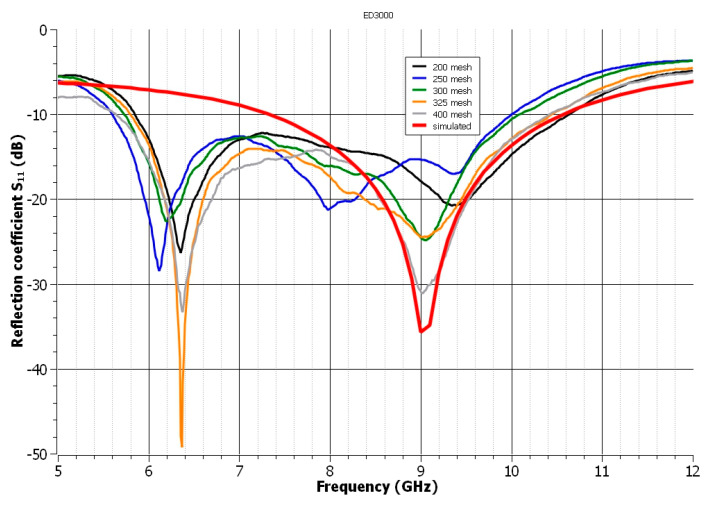
The influence of the mesh of the used screens on the reflection coefficient of antenna—paste ED3000.

In the case of the second analyzed polymeric paste, ED3000, the best result was obtained when a screen with a mesh size of 400 was used. The realized antenna’s bandwidth was increased to 3.8 GHz from 6.6 to 10.4 GHz. On the other hand, as illustrated in Figure 4, the mesh of the used screens had the biggest impact on the antenna’s reflection coefficient. Meshes 200 and 250 cause the shift in the resonance frequency and an insufficient reflection coefficient. Meshes of up to 300 cause the UWB antenna’s resonant frequency to stabilize and the reflection coefficient to decrease in accordance with the simulated curve. The manufacturer recommends a mesh size range of 200 to 325 for stainless steel screens. The results demonstrate that increasing the mesh size also improves the quality of the printed structure in this context.

**Figure 5:**
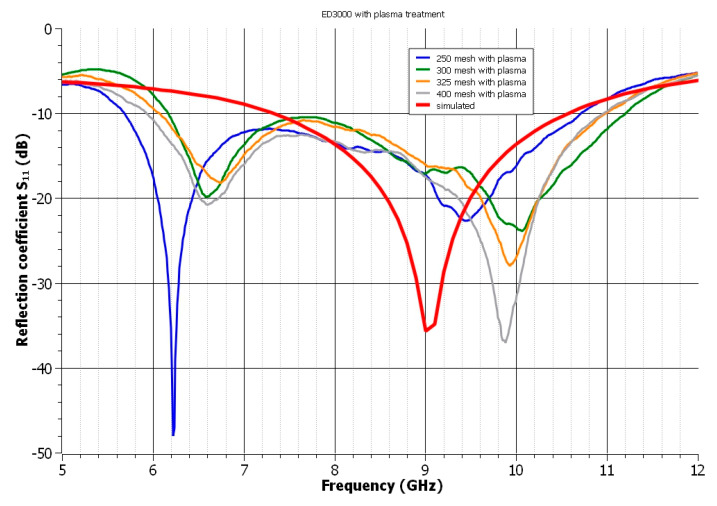
The influence of the mesh of the used screens on the reflection coefficient of the antenna after the plasma treatment—paste ED3000.

**Figure 6:**
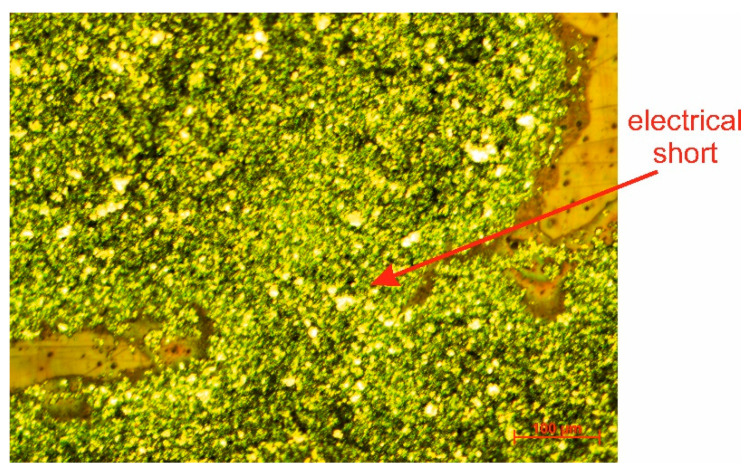
Electrical short caused by the plasma treatment in the case of the paste ED3000 (200 mesh).

Plasma treatment of the polymeric paste ED3000 has the same effect as it does with the paste XZ250, essentially changing the resonance frequency of the UWB antenna (Figure 5). As illustrated in Figure 6, when the screen with 200 mesh was plasma-treated, an electrical short occurred.

**Figure 7:**
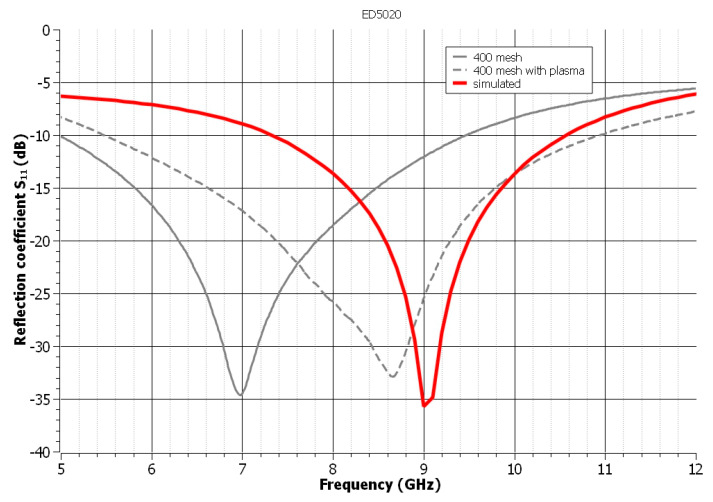
The influence of the mesh of the used screens on the reflection coefficient of the antenna—carbon-based paste ED5020.

The future of graphene-based materials is bright due to their beneficial properties and, most importantly, their ecological origin. Graphene is an organic material, and its use in combination with polymeric insulators enables green electronics [59,60]. Graphene has a lower conductivity than printed metallic inks or pastes based on silver particles, which is the most frequently used conductor material in the fabrication of flexible antennas [11]. Nevertheless, graphene-based structures have a number of advantages, including chemical and mechanical stability, low cost, fatigue resistance, stability in harsh environments, and bending resilience, making them an ideal candidate for flexible antennas [61]. The behavior of graphene compared to silver is significantly different. Graphene is a semi-metal with no band gap and extremely high carrier mobilities (up to 200,000 cm^2^V^−1^s^−1^) due to its unique two-dimensional honeycomb lattice [62,63], in comparison to silver (which forms the basic conductive component of the remaining screen-printing pastes), which is a metal with a typical band structure and carrier mobility of 50 cm^2^V^−1^s^−1^ at room temperature [64]. There is also a frequency dependence in graphene’s surface conductivity, but this is more evident at THz frequencies [62], which has not been investigated in the paper because of the UWB’s definition by FCC. As a result, the antenna was also realized using Electra’s screen-printing paste ED5020. The ED5020 screen-printing paste is developed for use on rigid substrates. Due to the difference in thermal expansion coefficients (TEC) between the paste and the polyimide substrate, only samples printed through the 400 mesh screen were investigated. As illustrated in Figure 7, the BW of the realized antenna is greater than that of the simulation. The BW is 4.42 GHz from 5 to 9.42 GHz before plasma treatment. After plasma treatment, the BW is 5.45 GHz, ranging from 5.5 to 10.95 GHz. The application of the plasma treatment results in an increase in the resonant frequency. As demonstrated in Figure 7, graphene-based materials are the most suitable alternative to conventional etching technologies. It is critical to keep in mind that by applying the plasma treatment, the screen-printing paste that is typically used on rigid substrates can also be used on flexible substrates.

By removing organic contaminants from the surface and attaching oxygen-containing molecules to it, the plasma treatment aims to convert a low-energy surface to a high-energy surface [65,66]. This results in the increased wettability of polymeric substrates and the spreadability of screen-printing pastes. If the paste spreads more widely on the surface, the radiation area becomes larger, and the feeding element becomes wider. As a result, the antenna’s resonance frequency and bandwidth are modified. As demonstrated in this paper’s experiments, plasma treatment leads to a change in the resonant frequency and an increase in the bandwidth of the realized UWB antenna in each investigated screen-printing paste.

**Figure 8:**
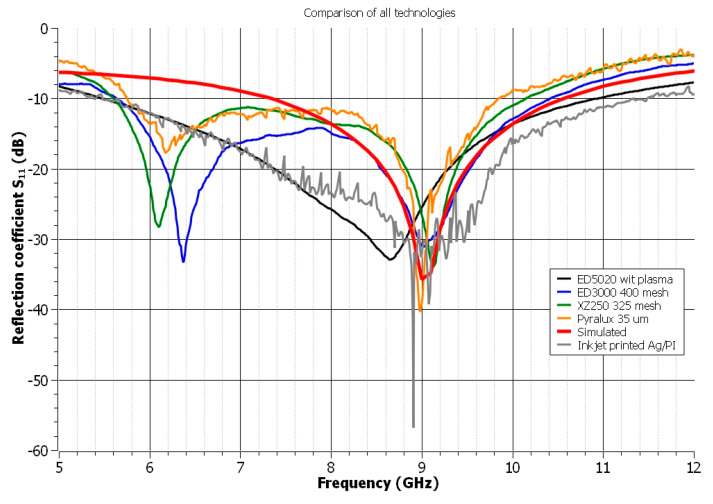
The reflection coefficient of all analyzed materials—selection of the best solutions.

**Figure 9:**
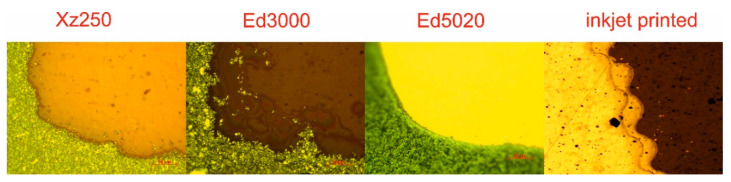
The edges of the realized UWB antenna by screen-printing with the 400 mesh and inkjet-printing technology.

The reflection coefficient of the materials chosen to provide the best solutions is illustrated in Figure 8. As is obvious, the inkjet-printed UWB antenna has the highest reflection coefficient. It is necessary to keep in mind that the principle of inkjet printing technology is based on a drop-by-drop system, which results in the printed structure’s regular edges [53], as illustrated in Figure 9. Smooth or regular edges are critical in the HF area due to the uniform wave reflection. Additionally, inkjet printing technology allows precise printing. This technology’s combination of these two advantages makes it the most suitable for the realization of UWB antennas [13,14]. Despite the shift in resonance frequency, graphene-based materials also provide an attractive solution for the implementation of UWB antennas due to their wide BW. However, in the case of the paste ED5020, it is critical to match the design to the paste’s low spreading characteristics or plasma treat the polymeric substrate’s surfaces. Both polymer-based screen-printing pastes containing silver particles are appropriate for realizing UWB antennas; nevertheless, it is imperative to analyze the effect of the screen mesh on the final quality of the UWB antenna. Polyimide substrates coated with a copper layer, called Pyralux, provide consistent quality in high-frequency applications. From a green electronics standpoint, this method is the least acceptable because of the hazardous wastes.

The antennas were fabricated using a variety of conductive materials (the silver-based pastes XZ250 and ED3000, as well as the nano-ink JP-6n, the graphene-based screen-printing paste ED5020, and polyimide film coated with copper—Pyralux). The conductivity of the materials used had no effect on the scattering parameter of the UWB antenna. As a consequence, this influence was not investigated in this paper.

The simulation was performed on a 50 µm-thick polyimide substrate with a dielectric constant of 3.4 and a homogenous conductive layer. It is important to keep in mind that the experiments compare three different manufacturing technologies, two of which are based on particle sintering or curing, which results in an inhomogeneous structure. The process of the sintering of particles lies in a connection of particles by necks into the conductive structure, whereas the final structure consists of the conductive necks and polymeric material of which the cavities around the necks are filled [67]. This polymeric material and its particle dispersion have a direct effect on how the transmitted signal scatters [59]. The behavior of the electronics structure (antenna, filter, etc.) varies depending on the sintering conditions of the particles obtained in pastes [68]. Additionally, screen-printing technology entails the development of the exposure mask, screen, and printing process. All of these factors lead to imperfections, and the designed structure will probably differ slightly from the realized structure. As a result, in some circumstances, the measured bandwidth of the reflection coefficients matches well with the simulation. However, except for the material ED5020, the measured antennas’ resonance frequencies match the simulation. The resonance frequency is 300 MHz lower in this case.

The homogeneity, thickness, and roughness of the layers do influence the signal transmission loss and, in this case, the UWB antenna’s reflection coefficient [69,70,71]. The results presented in this paper proved the effect of these parameters on the antenna behavior. The lower thickness of the printed layers is achieved by increasing the mesh size of the screen-printing pastes used, which offers a predisposition to precise edges in critical areas of the antenna. On the other hand, the thinner conductive layer more closely replicates the surface of the polymeric substrate, resulting in inhomogeneity. During the deposition process, plasma treatment has an effect on the surface properties of the substrates. In both cases, depending on the mesh, the resonance frequency of the UWB antenna was shifted up to the higher frequency using the polymeric pastes XZ250 and ED3000.

As stated previously, the edges of the realized structure are critical in HF applications. As a consequence, Figure 9 shows microscopic views of the critical part of the UWB antenna. In a comparison of screen-printed polymeric pastes, the polymeric paste XZ250 achieved the smoothest edges. The more disheveled edges were obtained with the ED3000 paste. Uneven edges can operate as a source of radiation and affect the final quality of the UWB antenna. A partial solution to this problem is to apply plasma treatment to the surfaces of polymeric substrates. Plasma treatment smooths the edges and partially eliminates the irregularities. On the other hand, plasma treatment causes the screen-printing pastes to spread more widely. The most refined edges were obtained with the carbon-based paste ED5020. The results demonstrate the potential of polymeric and graphene-based materials for the fabrication of UWB antennas.

The viscosity of all used pastes was measured as one of the rheology parameters using a HAAKE PK-100 with the measuring system M5 (maximum torque 4.9 Ncm, speed range 0.05 to 500 rpm) to support the understanding of screen-printed sample results. Rotovisco RV 20 and programmer PG 242 were used to ensure operation controls during the measurement, and an oil system HAAKE C with a regulator HAAKE F3 was used to regulate the temperature. A cone and plate system with a cone angle of 1° was used. Before the screen printing, the viscosity of the ED3000 and XZ250 pastes was not modified, however, the viscosity of the ED5020 paste was decreased using the Electrareducer ER7. The viscosities of all thick-film pastes used for screen printing at 25 °C, and three different shear rates (D = 30 s^−1^, 150 s^−1^, and 300 s^−1^), are presented in Table 2.

The measurements clearly show that the pastes had significantly varied viscosities. The waviness of the printed antenna’s edges (Figure 9) is the smallest for the highest viscosity paste (ED5020), and the shape of the pattern in the screen can be reproduced more precisely. In comparison, the printed edges of the pastes with a lower viscosity (XZ250 and ED3000) had a low quality. Together with the plasma treatment, which resulted in increased paste spreading, there was a more obvious change in antenna dimension and, as a result, in the final antenna parameters when compared to the simulation. As a result of this, it can be concluded that better results were obtained with ED5020 paste due to its rheological properties and better reproduction of the designed antenna’s dimensions.

## 4. Conclusions

This article discussed the suitability of polymer thick-film technology for the fabrication of UWB antennas. The paper presents a comparison of conventional technology for copper film etching, inkjet printing, and polymer thick-film screen printing in the context of realizing a UWB antenna. The result showed a significant influence of the screen mesh on the final quality of the UWB antennas and offered ways for achieving the highest possible reflection coefficient for the realized antennas. According to the results achieved in this paper, the optimal technology for realizing UWB antennas is inkjet printing, due to the precise printing and homogenous inequalities of the printed structures’ edges. Due to the low speed, the high cost of nanomaterials, and the difficulty of technological steps, inkjet printing technology is more suited for prototyping than rapid and large production. Therefore, screen printing was considered as a possible substitute for the methods mentioned. According to the findings in this research, there is a significant influence of electronics technology, as well as the circumstances of preparation and quality of the materials chosen, on the final quality of UWB antennas. The combination of graphene-based screen-printing technology and a polyimide substrate is one of the best material combinations for UWB antennas. This combination provides a promising ecological solution while retaining the UWB antenna’s reflection coefficient. Graphene is a low-cost material that can be processed at low temperatures and is therefore suitable for high-frequency applications. The surface of polymeric substrates can be plasma treated to change the characteristics of graphene-based pastes. Additionally, this article supports the concept of green electronics by demonstrating the possibility of realizing UWB antennas using new and modern materials rather than standard technology.

## Figures and Tables

**Table 1:** The electrical, thermal, and mechanical properties of the most commonly used polymeric substrates [45,46,47,48].

Substrate	Dielectric Properties		Thermal Properties				Mechanical Properties	
	ε_r_ (-) 100 Hz to 1 MHz	tanδ (%) 100 Hz to 1 MHz	CTE (ppm/°C) 15 °C to 300 °C	Shrinkage (%) 30 min, 150 °C	T_g_ (°C)	T_m_ (°C)	Tensile Strength (Kpsi)	Tensile Modulus (Kpsi)
PTFE	2.1 to 2.72	0.02 to 0.25	250 to 275	1.5 to 3.0	120 to 130	320 to 330	3.9 to 4.1	50 to 90
PP	2.2	0.01	100 to 200	n/a	−20 to −5	165 to 175	2.6 to 3.2	280
PI	3.4 to 3.9	0.13 to 0.40	20	0.03 to 1.25	310 to 365	-	22 to 33	330 to 400
PET	3.0	0.20	19 to 20	0.5 to 1.1	70 to 80	245 to 265	25 to 40	280 to 580
PDMS	2.3 to 2.8	0.15 to 0.35	340	0.03 to 2.7	−130 to −120	−50 to −40	0.25 to 1.3	0.522 to 0.126
LCP	2.9 to 3.14	0.25	17	0.03	n/a	n/a	29.0	327

**Table 2:** Measured viscosities of ED3000, XZ250, and ED5020 pastes at 25 °C.

Paste	Viscosity (Pa.s)		
	at D = 30 s^−1^	at D = 150 s^−1^	at D = 300 s^−1^
ED3000	3.9	2.2	2.0
XZ250	7.0	6.1	5.5
ED5020	39.8	26.2	17.1

## Data Availability

The data presented in this study are available on request from the corresponding author.
